# Incidence and risk factors for complications after definitive skeletal fixation of lower extremity in multiple injury patients: a retrospective chart review

**DOI:** 10.12688/f1000research.14825.1

**Published:** 2018-05-18

**Authors:** Thananit Sangkomkamhang, Wilaiphorn Thinkhamrop, Bandit Thinkhamrop, Wongsa Laohasiriwong

**Affiliations:** 1 Faculty of Public Health, Khon Kaen University, Khon Kaen , 40002, Thailand; 2 Data Management and Statistical Analysis Center, Faculty of Public Health, Khon Kaen University, Khon Kaen, 40002, Thailand

**Keywords:** Trauma registry, emergency medical services, retrospective cohort study, surgery

## Abstract

**Background**: The management of multiple injuries is complex. Type and timing of treatment for lower extremity fractures is a controversial subject. Although many studies have demonstrated the safety and effectiveness of early treatment, others have suggested that early definitive stabilization may cause complications, especially with chest and head injuries. The aim of this study was to determine the complications and effects of timing of fixation, and investigate risk factors for complications in multiple injuries patients with lower extremity fractures.

**Methods**: A Retrospective chart review from Khon Kaen Trauma Registry between 2008 and 2015 were collected. All major complications were identified and collected for example acute respiratory distress syndrome (ARDS), acute kidney injury (AKI) and sepsis.  The time to definitive skeletal fixation from initial injury was identified and analyzed with multiple logistic regression.

**Results**: 1224 multiple injuries patients with lower extremity fractures were identified. The mean age was 34±19.5 years, 74.4% were male and 25.6% female. The mean time from initial injury to definitive operation was 55.7±53.9 hours. Complications occurred with 178 patients (14.5%), the most common of which were pneumonia, ARDS and AKI. After adjusting for sex, severity of injury, we found that the operation within 24-48 hours complication was 6.67 times less common than in the early treatment group (less than 24 hours) (95% CI: 3.03 to 10.00, P-value< 0.001).

**Conclusions**: About 15% of the multiple injuries patients with lower extremity fracture had major complications. The optimal time for definitive fixation in lower extremity fractures to reduce complications was within 24-48 hours. We found that if we operated too early (before 24 hours) or more than 48 hours after the injury it could increase the morbidity and mortality.

## Abbreviations

ARDS        Acute respiratory distress syndrome

AKI           Acute kidney injury

MOF         Multiple organ failure

AIS           Abbreviated Injury Score

ISS           New Injury Severity Score

GCS         Glasgow Coma Scale score

PE            Pulmonary embolism

DVT         Deep venous thrombosis

OR           Odds ratio

95% CI     95% confidence interval

## Introduction

Management of lower extremity injury in patient with multiple injuries, especially femoral fractures, is a complex situation. The debate on the optimal time to fixation is commonly over two strategies, 1) early definitive fixation where definitive fixation is within 24 hours after the injury, and 2) damage control orthopedics (DCO). The benefits of early definitive fixation are lower pulmonary complications, shorter length of stay and less morbidity ^1– 4^. On the other hand, some of evidences suggested that early fixation of long bones have more complications in patient with head and chest injuries ^5– 7^ and can induce inflammatory processes, hypoperfusion and “second hit injury” ^8– 10^.

DCO involves an initial skeletal stabilization for the patients with multiple injuries and an unstable condition, followed by delayed definitive fixation ^11– 14^. DCO has some advantages over early fixation in pulmonary function, pain relief ^14^ and prevents the complication of early definitive fixation from initiating an inflammatory process that may be followed by acute respiratory distress syndrome (ARDS), acute kidney injury (AKI) and multiple organ failure (MOF) ^15, 16^. Many sources support the benefits of DCO ^17^. A meta-analysis from Robinson however, showed a protective effect of early definitive fixation from pulmonary complications when compared with DCO ( RR 0.30,0.22–0.40 90% CI) ^17^. Further complicating the situation, data has also been reported indicating no difference in morbidity and mortality between early and late definitive fixation of long bones ^18^.

Sine there is inconclusive evidence for the optimal time to perform definitive fixation of long bones, we conducted this study to determine the complications and effects of timing of fixation and investigate risk factors for complications in multiple injury patients with lower extremity fractures.

## Methods

A retrospective cohort study using data from the Khon Kaen Trauma Registry conducted between January 2008 and November 2015, was performed. 

### Study participants

A total of 1,224 multiple injury patients with lower extremity fractures treated between 2008 and 2015 were reviewed. Complications were identified from medical records that mentioned ‘major complication’ for example pneumonia, ARDS, AKI, sepsis and multiple organ failure (MOF). Timing to definitive skeletal fixation of lower extremity from initial injury were identified and analyzed with multiple logistic regression.

The Khon Kaen Trauma Registry collected pre-hospitalization and hospitalization information from medical records (including injury condition, comorbidities, fracture type, treatment type, operations, complications and outcomes)

**Figure 1.**
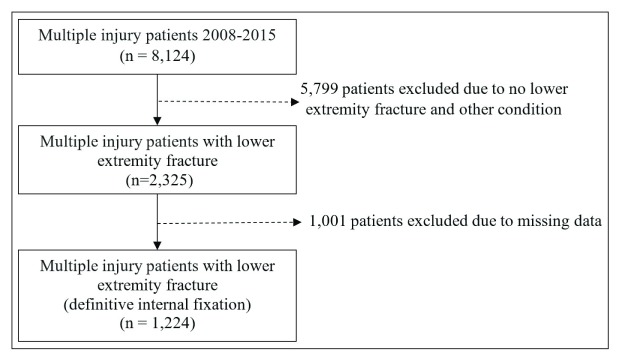
The flow of included patients from Khon Kaen Trauma Registry.

The inclusion criteria for this study were 1) closed or open diaphyseal fracture of the femur and tibia; 2) multiple injuries in at least 2 body region; 3) underwent a definitive treatment of the long bone fracture with internal fixation. The exclusion criteria were 1) patient with late admission or transferred from other hospitals; 2) pathological fracture caused by neoplasm or malignancy; 3) open fracture grade IIIB or IIIC classified by Gustilo *et al.* ^19^ or 4) inadequate information in the medical record ( Figure 1).

The information of covariates and potential confounding in The Khon Kaen Trauma Registry were collected to identify the association between time of treatment and complications. The potential confounders included age, sex, comorbidity, type of fracture, type of treatment, Abbreviated Injury Score (AIS) for each of the six anatomical body regions (head/neck, face, chest, abdomen, extremity/pelvis, and skin) ^20^, New Injury Severity Score (ISS) ^21^ and Glasgow Coma Scale score (GCS) on admission ^22^. In order to identify the association between time of definitive fixation of long bone and complications we divided patients with time of definitive fixation into three groups: 1) 24 hours or less, 2) between 24–48 hours, and 3) 48 hours or more from time of admission, based on cut-off points from previous studies ^23, 24^. Timing to definitive fixation was recorded in number of days and hours after admission.

### Assessment of outcome

The primary outcome analyzed was major complications, including pneumonia, pulmonary embolism (PE), ARDS, sepsis, deep venous thrombosis (DVT), AKI, MOF and mortality.

For diagnosis of ARDS, we defined it as an acute onset of bilateral pulmonary infiltrates on chest radiography and a PaO _2_:FiO _2_ 200 mm Hg for four days and had no evidence of pneumonia and cardiogenic pulmonary edema ^25, 26^. Acute kidney injury was defined by renal insufficiency which required hemodialysis ^27^. Multiple organ failure (MOF) was identified as failure of more than one organ system ^28^.

### Data analysis

Baseline characteristics and selected variables were analyzed using descriptive statistical method, categorical variables were presented as number and percent, continuous data were reported as mean, standard deviation (SD), median, minimum value and maximum value (Min: Max). The effect of time to definitive long bone fixation, potential confounding and major complications were analyzed by logistic regression. All significant factors were evaluated with logistic multivariate regression analysis to eliminate the effect of confounding factors. Results are reported with odds ratios (OR) and 95% confidence intervals (CIs). STATA 10 (Stata Corp., College Station, TX, USA) statistical software was used for analysis.

### Ethics statement

The current study utilized data from the database of Khon Kaen Trauma Registry between 2008 and 2015 which was conducted in a single hospital in Thailand. Permission for this purpose was obtained from the hospital before receiving an approval from the Institutional Review Board (IRB) of Khon Kaen University with the reference number of HE602122. The data analysis was performed after the permission gained from the registry and approval was obtained from the IRB.

## Results

The average age was 34.0 ±19.5 years. 910 patients (77.4%) were male and 314 (25.6%) were female. More than 50% of the patients (n= 785) were treated within 48 hours after admission. The average time to definitive fixation was 55.7±53.9 hours. The median time to definitive fixation was 35.0 hours (1.5–293.1). After categorizing the time to definitive fixation into three groups, it was found the highest proportion was in the group of delayed definitive fixation of more than 48 hours (38.7%). The mean Glasgow coma scale score was 14.3 ± 2.2. The mean Injury Severity Score was 8.5 ± 7.7. The mean Abbreviated Injury Scale was 2.6 ± 0.6. Femur fractures were the most common fractures (57.6%) ( Table 1).

**Table 1.** General characteristic of patients.

Characteristics	Number	Percent
**Age (year)**		
Children (<15)	151	12.3
Adult (15–49)	797	65.1
Old age (50–65)	172	14.1
Advance age (>65)	104	8.5
Mean ± SD	34.0 ± 19.5	
Median (Min: Max)	29 (1:94)	
**Gender**		
Male	910	74.4
Female	314	25.6
**Time to operate (hour)**		
<24	415	33.9
24–48	335	27.4
>48	474	38.7
Mean ± SD	55.7± 53.9	
Median (Min: Max)	35.0 (1.5: 293.1)	
**Glasgow Coma Scale (GCS)**		
<8	56	4.4
≥8	1168	95.4
Mean ± SD	14.3± 2.2	
Median (Min : Max)	15 (3:15)	
**Injury Severity Score (ISS)**		
<18	1089	89.0
≥18	139	11.0
Mean ± SD	8.5 ± 7.7	
Median (Min : Max)	5 (0.3:41)	
**Abbreviated Injury Scale (AIS)**		
<3	544	44.5
≥3	679	55.5
Mean ± SD	2.6 ± 0.6	
Median (Min : Max)	3 (1:5)	
**Body Region (BR)**		
Head/Neck	391	40.0
Face	11	0.9
Thorax chest	13	1.1
Abdomen and pelviccontents	28	2.3
Extremities and pelvic girdle	797	62.7
External and body surface	13	1.1
**Type of fracture**		
Femur	705	57.6
Tibia	519	42.4
Multiple fracture	767	62.7
**Type of fixation**		
Nail system	293	23.6
Plating system	783	64.4
External fixator	148	12.1

Overall, complication were found in 178 patients (13.4%). Most of the complications were pulmonary complications, specifically, pneumonia (6.7%) and ARDS (5.8%). Other complications were acute kidney injury (2.2%) and sepsis/MOF (0.9%). Patients with definitive fixation performed between 24–48 hours had fewer major complications than other groups as shown in Table 2. Pneumonia was found in 82 patients in total, 41 of which had definitive fixation more than 48 hours after injury, 37 in <48 hours group, and only 4 patients in the group were the fixation was performed between 24–48 hours after injury (6.7 per 100 people per year (PPY) (10.3–24.1;95%CI). A similar pattern was found in AKI and ARDS with an incidence of 2.2 per 100 PPY and 5.8 per 100 PPY respectively.

**Table 2.** The incidence of complications.

Time	Number, Incidence of complications, and 95% CI of incidence				
	Major complications			Severe complications	*Any complications
	Pneumonia	AKI	ARDS	Sepsis/MOF	
**Overall**	82 6.7 (4.1 to 10.3)	27 2.2 (1.4 to 2.6)	71 5.8 (4.5 to 6.3)	26 0.9 (0.5 to 1.0)	178 14.5 12.5 to 19.2)
**< 24 hrs**	37 8.9 (6.1 to 11.7)	11 2.7 (1.1 to 4.2)	39 9.4 (6.6 to 12.2)	2 4.80 (1.9 to 11.5)	87 21.0 (17.0 to 24.9)
**24 – 48 hrs**	4 1.2 (0.02 to 2.4)	1 0.3 (0.02 to 0.8)	8 2.4 (0.7 to 4.0)	2 6.0 (2.3 to 14.2)	13 3.9 (1.8 to 6.0)
**> 48 hrs**	41 8.7 (6.1 to 11.2)	15 3.2 (1.6 to 4.7)	24 5.1 (3.1 to 7.0)	3 6.30 (0.8 to 13.4 )	78 16.5 (13.1 to 19.8)

*Acute respiratory distress syndrome (ARDS), acute kidney injury (AKI), multiple organ failure (MOF), hrs = hours

The data in Table 3 shows the association between major complications and time to definitive fixation together with other covariates. Time to definitive fixation between 24–48 hours had a statistical significant effect on decreasing the risk of major complications. (OR=0.15; 95% CI: 0.08–0.27; p-value = <0.001) compared with other groups. Factors that significantly increase the risk of major complication are being over 65 years of age (OR=2.36; 95% CI: 1.20–4.67; p-value = 0.010), Glasgow Coma Scale (GCS) of more than 8 (OR=0.07; 95% CI: 0.04–0.14; p-value = <0.001), an Injury Severity Score (ISS) more than 18 (OR=6.24; 95% CI: 4.10–9.50; p-value = <0.001). The Abbreviated Injury Scale (AIS), abdominal injury and lower extremities injury are also significantly associated with increased risk of major complications ( Table 3).

**Table 3.** The bivariate analysis of covariates with time to operate and treatment group Crude odds ratio (Crude OR).

Factor	Number	% Complication	Crude OR	95% CI	p-value
**Time to operate (hours)**					
<24	415	20.9	1		
24–48	335	3.9	0.15	0.08-0.27	<0.001
>48	474	16.5	0.74	0.53-1.04	0.085
**Age increase with 1 year**			1.01	1.02-1.03	0.007
**Age (year)**					
<15	151	11.2	1		
15–49	797	14.1	1.23	0.75-2.22	0.360
50–65	172	14.5	1.34	0.69-2.59	0.380
>65	104	23.1	2.36	1.20-4.67	0.010
**Gender**					0.620
Male	910	14.8	1		
Female	314	13.7	0.91	0.63-1.32	
**Glasgow Coma Scale (GCS)**					<0.001
<8	56	65.3			
≥8	11167	11.6	0.07	0.04-0.14	
**Glasgow Coma Scale increase 1 score**			0.73	0.69-0.77	<0.001
**Injury Severity Score increase 1 score**			1.07	1.05-1.09	<0.001
**Injury Severity Score (ISS)**					<0.001
<18	1115	11.6			
≥18	109	45.0	6.24	4.10-9.50	
**Abbreviated Injury Scale (AIS) increase 1 score**			3.30	2.52-4.35	<0.001
**Abbreviated Injury Scale (AIS)**					<0.001
<3	544	8.5			
≥3	679	19.4	2.61	1.83-3.73	
**Body Region (BR)**					
Head/Neck	391	22.3			
Face	11	9.1	0.35	0.04-2.76	0.320
Thorax chest	13	69.2	7.86	2.36-26.1	0.001
Abdomen and pelvic contents	28	64.3	6.29	2.8-14.12	<0.001
Extremities and pelvic girdle	767	8.2	0.31	0.22-0.44	<0.001
External and body surface	13	0			
**Type of fracture**					
Tibia	519	10.4	1		
Femur	705	17.6	1.83	1.31-2.58	<0.001
Multiple fracture	358	20.8	3.39	2.35-8.62	<0.001
**Type of fixation**					
Nail system	24	10.12	1		
Plating system	45	13.45	1.23	0.75-2.22	0.360
External fixator	89	15.34	1.34	0.69-2.59	0.380

Multivariable analysis, with multiple logistic regression and adjusted for potential confounders; GCS, AIS BR and type of fracture, found that the risk factors that are associated with major complications were time to definitive fixation being less than 24 hours, being aged more than 65 years and an ISS of more than 18 ( Table 4). Receiving definitive fixation between 24–48 hours following injury led a statistical significant decrease in risk of major complication (Adjusted OR=0.18; 95% CI: 0.10–0.33; p-value = <0.001) when compared with those who received that treatment lower than 24 hours. Those 65 years and older had a statistical significant increased risk of major complication (Adjusted OR=3.3; 95% CI: 1.6–6.5; p-value = <0.001). An Injury Severity Score (ISS) of more than 18 causes a statistical significant increase risk of major complication (Adjusted OR =5.90; 95%CI = 3.80-9.18; p – value = <0.001)

**Table 4.** The multivariable analysis of the time to operate and complication after adjusted for age and injury severity score (Adjusted OR).

Factor	Number	% Complication	Crude OR	Adjusted OR	95%CI	p-value
**Time to operate (hour)**						
<24	415	20.9	1			
24-48	335	3.9	0.15	0.18	0.10-0.33	<0.001
>48	474	16.5	0.74	0.85	0.53-1.05	0.100
**Age (year)**						
<15	151	11.2	1			
15-49	797	14.1	1.23	1.43	0.80-2.55	0.030
50-65	172	14.5	1.34	1.65	0.81-3.33	0.170
>65	104	23.1	2.36	3.30	1.56-6.5	<0.001
**Injury Severity Score (ISS)**						
>18	1115	11.6	1			
≥18	109	45.0	6.24	5.90	3.80-9.18	<0.001

Raw data obtained from the Khon Kaen Trauma Registry between 2008 and 2015. Click here for additional data file. Copyright: © 2018 Sangkomkamhang T et al. 2018 Data associated with the article are available under the terms of the Creative Commons Zero "No rights reserved" data waiver (CC0 1.0 Public domain dedication).

## Discussion

Earlier studies have demonstrated the advantage of early definitive treatment of long bone fractures, especially in femoral fractures, over delayed fixation ^5, 6^. The DCO developed as a treatment option for multiple injury patients with long bone fractures, combines the advantages of early fixation and decreases the physiologic and inflammatory process after the major orthopedic procedure ^15, 29^. Despite DCO being associated with a shorter operative time and less blood loss than definitive fixation (IMN or plating), the retrospective study of 97 severe multiple injured patients with ISS more than 25 showed no difference in complication of ARDS and MOF when compared with early definitive fixation ^14^.

We found that delayed treatment of more than 48 hours after admission significantly increased the risk of complications compared with treatment within 24 hours or 24–48 hours. In some situations, the definitive treatment may be delayed more than two weeks after admission due to an unstable condition and is associate with further complications. These findings reflected the effect of the timing of definitive long bone fixation, especially in femoral shaft fractures in patients with multiple trauma, and care should be taken to avoid delay of treatment of more than the 48 hours from admission, similar to the study of Morshed *et al* ^23, 30^. In a large cohort study among multiple injury patients with an ISS of more than 15 with femoral shaft fractures where they studied the effect of timing of definitive fixation of femoral shaft fractures, they showed an increased length of stay for patients treated within 48 to 120 hours compared with other groups, especially in patients with chest trauma (AIS > 2). This study found that patients treated within 24 hours have lower length of stay ^23^. In our study, we founding performing definitive fixation 24 to 48 hours after admission to the hospital, has improved outcomes and survival rates.

In the study of multiple injury patients with femoral shaft fractures (ISS >18) definitive fixation between 2 to 4 days after injury was associated with higher inflammatory conditions and an increased rate of multi-organ failure compared with fixation 5 to 8 days after the injury ^10^. Other studies, however, have shown different results, with higher mortality when the operation is performed within the 2 to 5 day after injury; both of these resulted in high morbidity. Our study results found that performing fixation within 24–48 hours decreased the complications ^29^.

Many studies have supported this evidence that patients with multiple injuries namely head injuries ^31^, chest injuries ^2^ or abdominal injuries ^30^ have higher morbidity and a higher risk of complications. For multivariate analysis with associated severe head injury, chest injury, abdominal injury and lower extremity injury, especially femoral fractures, after adjusting for confounding, show a decreased morbidity rate if fixation is performed within 24 to 48 hour.

In our study, for patients with an unstable conditions who received early definitive fixation within 24 hours had increased inflammation which has the effect of increasing mortality rate ^32, 33^.

A study on early fixation of femoral shaft fractures (less than 24 hours) with a hypoperfusion state (serum lactate, > 2.5 mmol/L) demonstrated a similar number of postoperative complications to our study.(8) Multiple injury without appropriate resuscitation produces a hypoperfusion state and increases the inflammatory response leading to end-organ injury ^34^. Damage control orthopaedics (DCO) involves temporary fixation of the fracture until resuscitation of the patient is adequate and patients are stable for definitive fixation ^15, 35, 36^. For this study we found the benefits of delaying definitive long bone fixation for at least 24 hours until patients were stable and then performing the definitive fixation within 24–48 hours after admission to prevent second hit injury.

The retrospective chart review has limitation were selection bias and information bias. Variation in the time from the injury scene to hospital, lack of data for patients transferred from other hospital, the accurate time to operate?, and in some cases the data for DCO being incomplete contribute to information bias. This study has no investigation of blood chemistry, inflammatory mediators due to the fact it was a retrospective review. Complex femoral fractures, especially in proximal femoral fractures and distal femoral fractures, are not be clearly identified as a which may be lead to delayed fixation due to the fracture configuration and not by patient’s condition, which may affect the data. Furthermore there was a large number of patients who were transferred from other hospitals and therefore were not included in the study.

Despite many limitations, our study has a large sample size of patients with multiple injuries and lower extremity fractures. The data has been collect from the Khon Kaen Trauma Registry, a referral and level I trauma center. The large cohort size supports the generalizability of our findings. The Khon Kaen Trauma Registry also has significant data on potential confounders, which was used for analytical multivariate methods.

## Conclusion

Nearly 20% of multiple injuries patients with lower extremity fractures had major complications. We found that the timing of definitive fixation of lower extremity fracture (especially with femoral fracture) in multiple injury patients is associate with major complications.

The optimal time for definitive skeletal fixation in lower extremity fractures with the least complications based on our analysis appears to be within 24–48 hours. Performing operation too early (before 24 hours) or too late (after 48 hours) is associated with an increase in complication rate and should be considered in patient management for improved outcome.

The results support a delayed or “damage control orthopedics” management over definitive fixation of long bone fracture in multiple injuries patients. In patients who are unstable, adequate resuscitation at least 24 hours to 48 hours before undergo definitive fracture fixation is necessary, due to the systemic inflammatory response and to avoid “second hit” from a major surgical procedure. The trauma center hospitals should manage their resources to guarantee time to definitive fixation and proper management of multiple injuries patients. 

## Data availability

The data referenced by this article are under copyright with the following copyright statement: Copyright: © 2018 Sangkomkamhang T et al.

Data associated with the article are available under the terms of the Creative Commons Zero "No rights reserved" data waiver (CC0 1.0 Public domain dedication). 

Dataset 1: Raw data obtained from the Khon Kaen Trauma Registry between 2008 and 2015. 10.5256/f1000research.14825.d203372 ^37^
