# Supplementary material for: Incidence and risk factors for complications after definitive skeletal fixation of lower extremity in multiple injury patients: a retrospective chart review
Source: F1000Res. 2018 May 18;7:612. [Version 1] doi: 10.12688/f1000research.14825.1 (PMC5981188; doi:10.12688/f1000research.14825.1)
Supplement: Peer review history

# Peer review: Referee response for version 1

**DOI:** 10.5256/f1000research.16136.r34165

**Published:** 2018-05-31

**Authors:** Piya Kiatisevi

**Affiliations:** 1 Orthopaedic Oncology Service, Institute of Orthopaedics, Lerdsin General Hospital, Bangkok, Thailand

The authors reviewed large number of multiple injuries patients with lower extremity fracture and, in the abstract, concluded that the optimal time for definitive fixation to reduce complications was within 24-48 hours.

1. However, the authors did not clarify the reason for the delay surgery which could be unstable patient condition or operation room availability or other reasons. Complications in these groups could be different and affected the patient outcome. Do we really need to speed up the improvement process of the patient condition to get fracture fixed in 24-48 hours?
2. Can we conclude from this paper that definite fracture treatment before 24 hours is not recommended? For patients whose conditions are stable before 24 hours, should we wait until we get into 24-48 hours period to reduce the inflammatory process?
3. For the conclusion “optimal time for definitive fixation to reduce complications was within 24-48 hours”, are the severity of the injuries, type of injuries, pre-operative conditions of patients, fracture configuration and operative time in this group similar when compare to those of <24 hours and > 48 hours groups? Should the authors do subgroup analysis on this point?
4. The percentage of multiple injuries patients with lower extremity fracture who had major complications in the abstract is not equal those on the main manuscript, please correct.

I have read this submission. I believe that I have an appropriate level of expertise to confirm that it is of an acceptable scientific standard.

# Peer review: Referee response for version 1

**DOI:** 10.5256/f1000research.16136.r34168

**Published:** 2018-05-29

**Authors:** Saradej Khuangsirikul

**Affiliations:** 1 PMK Hip & Knee Surgery, Department of Orthopedics, Phramongkutklao Hospital and College of Medicine, Bangkok, Thailand

As the authors mentioned, selection bias was an issue. Type, classification, and fracture geometry, those were not recorded, also the important factors associated with postoperative complications. 

 Different experience of multiple surgeons should be mentioned for the information bias. 

 The reasons of delayed definitive operations should be classified, such as, patient condition, surgeon intension, randomisation, or hospital facilities etc.

 Exactly, "time from the injury scene to definitive fixation" is more concerned duration and more appropriate than "time from admission".

I have read this submission. I believe that I have an appropriate level of expertise to confirm that it is of an acceptable scientific standard.
